# Sorafenib Chemosensitization by Caryophyllane Sesquiterpenes in Liver, Biliary, and Pancreatic Cancer Cells: The Role of STAT3/ABC Transporter Axis

**DOI:** 10.3390/pharmaceutics14061264

**Published:** 2022-06-14

**Authors:** Silvia Di Giacomo, Marco Gullì, Roberta Facchinetti, Marco Minacori, Romina Mancinelli, Ester Percaccio, Caterina Scuderi, Margherita Eufemi, Antonella Di Sotto

**Affiliations:** 1 Department of Physiology and Pharmacology “V. Erspamer”, Sapienza University of Rome, P.le Aldo Moro 5, 00185 Rome, Italy; marco.gulli@uniroma1.it (M.G.); roberta.facchinetti@uniroma1.it (R.F.); ester.percaccio@uniroma1.it (E.P.); caterina.scuderi@uniroma1.it (C.S.); 2 Department of Biochemical Science “A. Rossi Fanelli”, Sapienza University of Rome, P.le Aldo Moro 5, 00185 Rome, Italy; marco.minacori@uniroma1.it (M.M.); margherita.eufemi@uniroma1.it (M.E.); 3 Department of Anatomical, Histological, Forensic and Orthopedic Sciences, Sapienza University of Rome, P.le Aldo Moro 5, 00185 Rome, Italy; romina.mancinelli@uniroma1.it

**Keywords:** β-caryophyllene, β-caryophyllene oxide, hepatocarcinoma, pancreatic cancer, cholangiocarcinoma, potentiation, sorafenib, ABC transporters, STAT3, chemoresistance

## Abstract

A combination of anticancer drugs and chemosensitizing agents has been approached as a promising strategy to potentiate chemotherapy and reduce toxicity in aggressive and chemoresistant cancers, like hepatocellular carcinoma (HCC), cholangiocarcinoma (CCA), and pancreatic ductal adenocarcinoma (PDAC). In the present study, the ability of caryophyllane sesquiterpenes to potentiate sorafenib efficacy was studied in HCC, CCA, and PDAC cell models, focusing on the modulation of STAT3 signaling and ABC transporters; tolerability studies in normal cells were also performed. Results showed that the combination of sorafenib and caryophyllane sesquiterpenes synergized the anticancer drug, especially in pancreatic Bx-PC3 adenocarcinoma cells; a similar trend, although with lower efficacy, was found for the standard ABC transporter inhibitors. Synergistic effects were associated with a modulation of MDR1 (or Pgp) and MRP transporters, both at gene and protein level; moreover, activation of STAT3 cascade and cell migration appeared significantly affected, suggesting that the STAT3/ABC-transporters axis finely regulated efficacy and chemoresistance to sorafenib, thus appearing as a suitable target to overcome drawbacks of sorafenib-based chemotherapy in hepato-biliary-pancreatic cancers. Present findings strengthen the interest in caryophyllane sesquiterpenes as chemosensitizing and chemopreventive agents and contribute to clarifying drug resistance mechanisms in HCC, CCA, and PDAC cancers and to developing possible novel therapeutic strategies.

## 1. Introduction

Liver, biliary tract, and pancreas are characterized by similar histological features arising from a common endodermal origin [1,2]. Accordingly, the function of acinar, hepatic, and ductal cells is regulated by common transcription factors [3,4], thus suggesting that similar mechanisms can drive carcinogenesis in these organs. Hepatocellular carcinoma (HCC), cholangiocarcinoma (CCA, including intra, extrahepatic and gallbladder cancers), and ductal adenocarcinoma (mainly pancreatic ductal adenocarcinoma or PDAC) are among the major malignancies occurring in the hepato-biliary-pancreatic system and are characterized by extremely poor prognosis, due to delay in diagnosis, tumor aggressiveness, lack of proper therapies, and chemoresistance to anticancer drugs [1,5]. Chemoresistance is a major impediment for the treatment of HCC, CCA, and PDAC cancers and is a consequence of multiple factors, including individual variability, tumor phenotype, and cell alterations [1,5,6].

The entire set of proteins contributing to the mechanisms of chemoresistance exploited by tumors to endure chemotherapeutic effects is defined as “resistome” [7]. Among them, the over-expression of ATP-binding cassette (ABC) transporters, responsible for drug efflux from cells, is often involved in multidrug resistance (MDR) and chemotherapy failure. In particular, MDR1 (P-glycoprotein), MRP1 (multidrug resistance-associated protein 1), and MRP3 (multidrug resistance-associated protein 3) were found to be highly overexpressed in biliary cancer [8,9], whereas MDR1, MDR3, MRP3, and MRP5 were linked to a poor prognosis in pancreatic and liver cancers [10,11]. MDR1 and MRP1 transporters have been found to be transcriptionally regulated by the signal transducer and activator of transcription protein STAT3, whose increased expression in most iCCA is correlated with worse prognosis [4,5]. Although the inhibition of ABC transmembrane proteins has been approached as a suitable strategy to overcome cancer resistance, their involvement in physiological detoxification processes suggests the need for more studies to better design possible modulating interventions and to reveal the mechanisms involved in their deregulation.

Current chemotherapeutic regimens for hepato-biliary-pancreatic cancers, usually sorafenib for treating unresectable HCC [11] or gemcitabine plus cisplatin and paclitaxel for biliary tract and pancreatic cancers [6,12], have only low survival benefits, despite MDR development and severe toxicity, which often hinder the continuation of therapy [6,12].

To overcome these drawbacks, some innovative pharmacological regimens have been proposed; among them, drug combination (co-administration of many drugs with various mechanisms and targets) allows the simultaneous blockage of distinct key molecules in the oncogenic signaling network, thus affecting both primary pathways involved in cancer cell proliferation and the activation of specific deregulated mechanisms, which help cancer cells to overcome the treatment injury [13]. Moreover, combination with chemosensitizing agents may also potentiate the anticancer drugs, thus achieving the expected pharmacological effect at low doses, with reduced risks of side effects and complications [14].

Looking for chemosensitizing agents has attracted great attention by researchers due to the noteworthy application of this strategy in adjuvant chemotherapy or in sensitizing resistant cancer cells; however, variability in cancer phenotype and the complex network carrying chemoresistance make it difficult to identify an ideal mechanism to be targeted for counteracting its development [15].

A number of natural substances have been found to be able to synergize in vitro anticancer drugs and to resensitize cancer cells, acting as chemosensitizers [16]. Among them, β-caryophyllene and β-caryophyllene oxide, two natural sesquiterpenes with a typical caryophyllane skeleton, widely occurring in plants, especially in essential oils (e.g., clove oil, apple mint oil, and hemp essential oil), seeds (e.g., *Piper nigrum* L.) and rhizomes (*Harpagophytum procumbens* DC), were shown to possess promising chemosensitizing properties, along with pleiotropic pharmacological activities, including antioxidant, antinflammatory, genoprotective, neuroprotective, and chemopreventive ones, along with a safe toxicity profile [15,17,18]. Moreover, they were found to be able to modulate multiple cascades involved in oxidative stress, inflammation, and cell survival, such as PI3K/Akt/mTOR/S6K1, NF-kB and STAT3 [15].

Our previous studies highlighted the abilities of caryophyllane sesquiterpenes to synergize doxorubicin and sorafenib in different cancer cell lines, likely affecting MDR1, MRP1, and MRP2 pumps [13,19,20,21]. Moreover, β-caryophyllene promoted apoptosis as a result of a complex ROS/H2AX/STAT3 modulation in Mz-ChA-1 cells while producing protective effects in normal cholangiocytes, likely being a dual-acting chemosensitizing and chemopreventive agent [21]. Synergistic effects of β-caryophyllene oxide towards sorafenib were also confirmed in a xenograft model of liver cancer [20]. More recently, we reported the potentiating effects of caryophyllane sesquiterpenes towards cannabidiol and harpagoside in hemp and devil’s claw extracts, respectively [18,22].

In line with this evidence, in the present study we investigated the chemosensitizing properties of β-caryophyllene and β-caryophyllene oxide in combination with sorafenib in human liver, biliary, and pancreatic cancer cells and the mechanisms, focusing on the interconnection between STAT3 cascade, involved in cell survival and growth, and ABC transporter function and expression. To this end, combination studies and efflux assays with specific fluorescent substrates (e.g., rhodamine 123 for MDR1, calcein acetoxymethyl ester for MRPs) have been performed; moreover, modulation of gene and protein expression of efflux pumps and STAT3 protein has been investigated. Tolerability of the combined treatment of sorafenib and caryophyllane sesquiterpenes in normal cholangiocytes, which represents an important goal to be achieved to overcome toxicity drawback of chemotherapy, has also been evaluated.

## 2. Materials and Methods

### 2.1. Chemicals

The chemicals β-caryophyllene (≥98.5% purity), β-caryophyllene oxide (95% purity), and verapamil hydrochloride (≥98.0% purity) were purchased from Merck Life Science S.r.l. (Milan, Italy), while sorafenib tosylate (≥99.0% purity) and MK571 (≥96.0% purity) were purchased from Santa Cruz Biotechnology, Inc. (Santa Cruz, CA, USA). Dulbecco’s Modified Eagle’s medium (DMEM), Roswell Park Memorial Institute (RPMI) 1640 culture medium, fetal bovine serum, buffer, and cofactors were from Aurogene S.r.l. (Rome, Italy). EtOH (100% *v*/*v*) was used to dissolve β-caryophyllene and β-caryophyllene oxide, while DMSO (100% *v*/*v*) was used for sorafenib tosylate, verapamil hydrochloride, and MK571. Solvents were used up to 1% *v/v* nontoxic concentration.

### 2.2. Cell Lines

Hepatoblastoma (HepG2), extrahepatic cholangiocarcinoma (Mz-ChA-1), and pancreatic adenocarcinoma (Bx-PC3) cells were exploited as experimental models. Moreover, noncancerous H69 intrahepatic cholangiocytes were included for tolerability studies [21]. HepG2 cells were a kind gift from Prof. F. Altieri (Dept. of Biochemical Sciences, Sapienza University of Rome), while Mz-ChA-1 and H69 were from Prof. G. Alpini (Indiana University School of Medicine, Indianapolis, IN, USA). Bx-PC3 were purchased from Interlab Cell Line Collection (IRCCS San Martino Policlinico Hospital, Genoa, Italy). The cell lines were grown using appropriate media and co-factors under standard conditions (37 °C and 5% CO_2_), according to previous studies [13,21,23]. The growth media were changed twice per week, as recommended by the suppliers. When the cells reached approximately 80% confluency, they were subcultured.

### 2.3. Treatment Schedule

**Figure 1:**
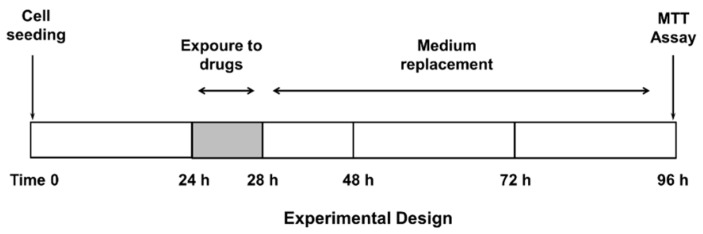
Treatment schedule chosen to assess the chemosensitizing properties of caryophyllane sesquiterpenes.

Confluent cells were treated with the test substances, both alone or in combination with the anticancer drug, for 4 h, then washed and incubated for a further 72 h (Figure 1), according to previously published protocols [20]. This treatment schedule has been chosen to highlight the potential effect of β-caryophyllene and β-caryophyllene oxide on ABC transporters and reduce a possible sorafenib effect due to diffusional accumulation after long exposure to this drug [20]. The test substances were assayed within the following ranges: sorafenib, 0.1–500 µg/mL (corresponding to 0.2–1076 µM); verapamil, 1–500 µg/mL (corresponding to 2–1099 µM); MK571, 1–500 µg/mL (corresponding to 2–932 µM); β-caryophyllene, 1–250 µg/mL (corresponding to 5–1223 µM); and β-caryophyllene oxide, 1–250 µg/mL (corresponding to 5–1134 µM). Suitable vehicle controls were also included.

### 2.4. Cytotoxicity Assay

The effect of the treatments on cell viability was measured by the MTT assay as previously described [13]. A higher than 30% reduction of cell viability was considered as a significant cytotoxic effect [23].

### 2.5. Combination Assay and Analysis of Sesquiterpene-Drug Interactions

To evaluate the potential chemosensitizing properties, the cytotoxicity of sorafenib (0.1–250 µg/mL corresponding to 0.2–538 µM) in combination with a nontoxic concentration of β-caryophyllene and β-caryophyllene oxide (10 µg/mL corresponding to 50 µM), selected in preliminary experiments, was tested by MTT assay [13]. The combination of sorafenib with a nontoxic concentration of the positive controls, i.e., verapamil and MK571 (1 µg/mL corresponding to 2 µM), was also tested. The type of interaction (synergistic, additive, or antagonistic) among sorafenib and test substances or positive controls was evaluated by measuring the reversal ratio value (RR), combination index (CI), and isobolographic analysis (IB), as previously described [21].

### 2.6. ABC-Mediated Drug Efflux Assay

The ability of β-caryophyllene and β-caryophyllene oxide to inhibit the activity of Pgp and MRP1/2 transporters was measured according to previously published methods [13,20] with slight modifications. Briefly, confluent cells (2 × 10^4^ cells/well) were subjected to a 2 h pre-treatment with the tested sesquiterpenes (10 μg/mL corresponding to 50 μM) or positive controls verapamil and MK571 (1 μg/mL corresponding to 2 μM), which represent the standard Pgp and MRP1/2 inhibitors respectively; then, the fluorescente probes rhodamine 123 (1 μM, for MDR1) and calcein acetoxymethyl ester (0.05 μM for MRP1 and MRP2) were added. Fluorescence was measured at excitation and emission wavelengths of 485 nm and 535 nm for both probes, using a Cytation 1 Cell Imaging Multimode Reader (Biotech, Santa Clara, CA, USA). Results were normalized to viable cells and expressed as percentage of the negative control.

### 2.7. Wound Healing Assay

To evaluate the effect of caryophyllane sesquiterpenes, both alone and in combination with sorafenib, on the cell migration rate, the wound healing assay was performed. Bx-PC3 cells were seeded in 24-well plate at a density of 2.5 × 10^5^ cells/well. After 24 h, the wound was created by scratching the well bottom with a 100 μL pipette tip, then the cells were treated under the scheduled conditions and images were captured at 0, 4, and 72 h using a Cytation 1 Cell Imaging Multi-Mode Reader. The wound area was calculated by using the ImageJ software (ImageJ 1.52n, National Institutes of Health, Bethesda, MD, USA). Results were expressed as wound area percentage with respect to that of the same well at zero time. The positive controls were also included.

### 2.8. Western Blotting Analysis

Confluent cells were seeded in 6-well plates (5 × 10^5^ cells/well), treated as described above, then harvested by centrifugation and washed in PBS. After lysis with a suitable buffer, the Western blotting analysis was carried out, according to previously published methods [21]. The primary antibodies anti-phospho-STAT3 (Tyr705) (rabbit antibody; 9145S from Cell Signaling Technology, EuroClone, Pero, Italy; dilution factor 1:2000) and anti-STAT3 total (mouse antibody; 9139S from Cell Signaling Technology, EuroClone, Pero, Italy; dilution factor 1:1000) were used, then staining with the appropriate alkaline phosphatase-conjugated secondary antibody was carried out. The intensity of protein bands was quantified by the ImageJ software (ImageJ 1.52n, National Institutes of Health, Bethesda, MD, USA). The levels of phospho(Tyr705)-STAT3 were normalized against total STAT3 ones.

### 2.9. Immunofluorescence Analysis

The analysis was performed according to previously published methods [13]. Confluent cells were seeded in 24-well plates (2 × 10^4^ cells/well), allowed to adhere overnight, then treated as described above. After treatment, cells were fixed in pure methanol and washed with 4% bovine serum albumin (BSA) and PBS + Tween 20 (PBS-T) for 1 h. Then, the primary antibodies against MDR1/ABCB1 (mAB #13342, Cell Signaling Technology, Danvers, MA, USA; dilution factor 1:400), MRP1 (sc-18835, Santa Cruz Biotechnology Inc., Santa Cruz, CA, USA; dilution factor 1:500), and MRP2 (bs-1092R, Bioss Inc., Woburn, MA, USA; dilution factor 1:200), followed by staining with specific secondary antibodies (dilution factor 1:800) and nucleic acid dye (Hoechst 33258, 1 µg/mL), were added. The expression and localization of Pgp, MRP1, and MRP2 pumps into cells were analyzed by using a Cytation 1 Cell Imaging Multimode Reader (Biotech, USA). Fluorescence intensity was determined by Gen5™ Microplate Reader and Imager Software 3.11, and normalized with respect to cell number.

### 2.10. Gene Expression Analysis by RT-qPCR

Gene expression was analyzed by RT-qPCR (Real-Time Quantitative Polymerase Chain Reaction), according to previous methods [24]; details about primers are shown in Table 1. The results of mRNA abundance for the targeted genes in each sample were normalized on the basis of GAPDH (Bio-Rad, Hercules, CA, USA) mRNA. Data are expressed as 2^−ΔCt^, calculated using the CFX Manager software (Bio-Rad).

### 2.11. Statistical Analysis

Data from at least three independent experiments, each one including at least three technical replicates per treatment, were pooled, analyzed by GraphPad Prism™ (Version 6.00) software (GraphPad Software, Inc., San Diego, California, USA) and expressed as mean ± standard error (SE). Significance of the response (*p* value < 0.05) with respect to control was evaluated by the one-way analysis of variance (one-way ANOVA), followed by Dunnett’s multiple comparison post-test or by Student’s *t*-test. The concentration–response curves were constructed using the Hill equation [20].

## 3. Results

### 3.1. Cytotoxicity of Sorafenib and Caryophyllane Sesquiterpenes in Human Hepato-Biliary-Pancreatic Cancer Cell Lines

**Figure 2:**
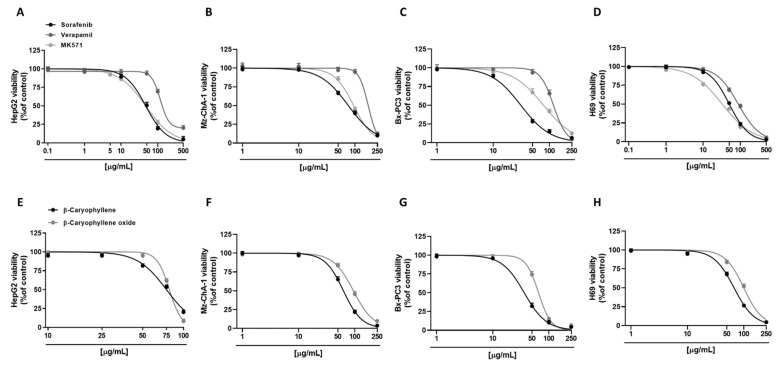
Cytotoxicity effects of sorafenib, verapamil, MK571, and caryophyllane sesquiterpenes β-caryophyllene and β-caryophyllene oxide in hepatoblastoma HepG2 (**A**,**E**), cholangiocarcinoma Mz-ChA-1 (**B**,**F**), and pancreatic adenocarcinoma Bx-PC3 cells (**C**,**G**), and noncancerous H69 cholangiocytes (**D**,**H**) after 4 h exposure and 72 h recovery time. Data displayed as mean ± SE of at least three independent experiments (*n* = 3).

Preliminarily, cytotoxicity of the tested compounds under the scheduled exposure protocol (i.e., a short 4 h exposure followed by a 72 h recovery time) was evaluated in all human cancer cell lines (i.e., hepatoblastoma HepG2, cholangiocarcinoma Mz-ChA-1, and pancreatic adenocarcinoma Bx-PC3 cells), in order to find the suitable concentrations to be used in the combination assays. As expected, the anticancer drug sorafenib was the most cytotoxic compound. In particular, in HepG2 cells, it exerted early signs of toxicity (approximately 10% inhibition of cell viability) at 10 µg/mL, achieving a maximum inhibition of 95% at the highest tested concentration of 500 µg/mL (Figure 2A). Conversely, Mz-ChA-1 cells were resistant to sorafenib; indeed, 50 µg/mL sorafenib lowered cell viability by about 30%, while the maximum cytotoxicity (about 90% inhibition of cell viability) was achieved at the concentration of 250 µg/mL (Figure 2B). Bx-PC3 cells were the most sensitive to sorafenib: a 70% inhibition of cell viability was induced by 50 µg/mL sorafenib, being almost completely inhibited at the higher tested concentrations (Figure 2C). The different behavior of sorafenib among cell lines was also confirmed by the IC_50_ values (Table 2), which were approximately 1.5- and 2.4-fold higher in HepG2 and Mz-ChA-1 cells with respect to Bx-PC3 ones.

By comparison, we also evaluated the cytotoxic effects of verapamil and MK571, which are known inhibitors of MDR1 (or Pgp) and MRP pumps, respectively [20,25]. In this regard, verapamil was highlighted as the least cytotoxic compound in all the cell lines; conversely, cytotoxicity of MK571 was comparable to that of sorafenib in HepG2 cells, producing a 50% lowering of cell viability at 50 µg/mL (Figure 2A). Verapamil exhibited a similar cytotoxicity profile in HepG2 and Bx-PC3 cells, inducing a 30% inhibition of cell viability at the concentration of 100 µg/mL, while almost a complete inhibition by about 80% and 96% was achieved at 500 µg/mL and 250 µg/mL in HepG2 and Bx-PC3, respectively (Figure 2A,C). As for sorafenib, Mz-ChA-1 cells were the most resistant to verapamil treatment: only a 5% inhibition of cell viability was induced at 100 µg/mL, whilst an 88% cytotoxicity was induced at 250 µg/mL (Figure 2B). MK571 produced early toxicity signs at 50 µg/mL in Mz-ChA-1 and Bx-PC3 cells (approximately 15% and 33% inhibition of cell viability, respectively), reaching approximately a 90% inhibition at 250 µg/mL (Figure 2B,C). Comparing the IC_50_, verapamil showed about 2.5-, 2.2-, and 3.8-fold higher values than those of sorafenib in HepG2, Mz-ChA-1, and Bx-PC3 cells, respectively (Table 2). Moreover, the IC_50_ value of MK571 was comparable to sorafenib in HepG2 cells, and 1.2- and 2.5-fold higher than that of sorafenib in Mz-ChA-1 and Bx-PC3 cells, respectively (Table 2).

Regarding caryophyllane sesquiterpenes, both compounds showed a similar cytotoxicity profile in HepG2 cells (Figure 2E), as corroborated by the IC_50_ values (Table 2). Conversely, in Mz-ChA-1 and Bx-PC3 cells, β-caryophyllene showed a higher cytotoxicity with respect to β-caryophyllene oxide by approximately 1.5- and 1.8-fold, respectively. In particular, in Mz-ChA-1 cells, both compounds exerted early signs of cytotoxicity at 50 µg/mL; however, while β-caryophyllene induced a 35% inhibition of cell viability, β-caryophyllene oxide reduced cell viability by 16% (Figure 2F).

For both compounds, the maximum inhibition (96% and 90%, respectively) was achieved at 250 µg/mL (Figure 2F). In Bx-PC3 cells, despite a 70% inhibition of cell viability induced by β-caryophyllene at 50 µg/mL, β-caryophyllene oxide reduced cell viability by only 30% (Figure 2G); both compounds induced the maximum 95% inhibition at 250 µg/mL (Figure 2G).

Comparing the IC_50_ values, those of β-caryophyllene and β-caryophyllene oxide were 1.6- and 1.7-fold higher than that of sorafenib in HepG2 cells. Conversely, in Mz-ChA-1 cells, β-caryophyllene displayed an IC_50_ value approximately 1.2-fold lower with respect to sorafenib; by contrast, that of β-caryophyllene oxide was 1.2-fold higher than the anticancer drug. Finally, in Bx-PC3 cells, IC_50_ value of β-caryophyllene was comparable to sorafenib, while that of β-caryophyllene oxide was 2.1-fold higher (Table 2).

Tolerability studies, carried out in H69 noncancerous cholangiocytes, highlighted that β-caryophyllene oxide was the least cytotoxic compound followed by verapamil, β-caryophyllene, sorafenib, and MK571 (Figure 2D,H). Comparing noncancerous H69 and cancerous Mz-ChA-1 cholangiocytes, β-caryophyllene, and β-caryophyllene oxide showed similar IC_50_ values with respect to the corresponding cancer cell line; conversely, the anticancer drug sorafenib and the positive controls verapamil and MK571 exerted a higher cytotoxicity in normal cells by approximately 1.5-, 1.9-, and 2.9-fold, respectively (Table 2).

Based on present results, the nontoxic concentrations of 10 µg/mL for both caryophyllane sesquiterpenes and 1 µg/mL for the positive controls verapamil and MK571, which produced slight or null reduction (lower than 10%) of cell viability in tumoral and normal cells, have been selected to be tested in the subsequent combination experiments with sorafenib.

### 3.2. Chemosensitizing Effects of Caryophyllane Sesquiterpenes in Combination with Sorafenib in Human Hepato-Biliary-Pancreatic Cancer Cell Lines

The chemosensitizing properties of caryophyllane sesquiterpenes have been investigated in combination with progressive concentrations of sorafenib; moreover, combinations of sorafenib with verapamil and MK571 were also tested to understand if the observed increase of sorafenib cytotoxicity could be related to the inhibition of ABC pumps. Indeed, these compounds are known inhibitors of Pgp and MRP1-2, which are involved in sorafenib resistance [20,25].

**Figure 3:**
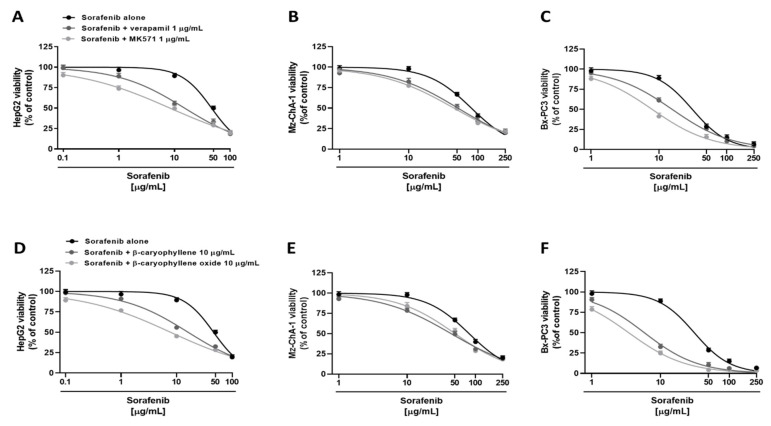
Cytotoxicity of sorafenib in combination with verapamil and MK571 (**A**–**C**), and the sesquiterpenes β-caryophyllene and β-caryophyllene oxide (**D**–**F**) in hepatoblastoma (HepG2), extrahepatic cholangiocarcinoma (Mz-ChA-1), and pancreas adenocarcinoma (Bx-PC3) cells after 4 h exposure and 72 h recovery time. Data displayed as mean ± SE of at least three independent experiments (*n* = 3).

Under our experimental conditions, both verapamil and MK571 were able to potentiate sorafenib. Particularly, in HepG2 cells, they determined an increase by about 8% and 23% of sorafenib cytotoxicity at the concentration of 1 µg/mL, and by about 34% and 40% at the concentration of 10 µg/mL, despite a null effect of the anticancer drug alone (Figure 3A). Overall, sorafenib IC_50_ was approximately 3- and 6-fold lower in the presence of verapamil and MK571, respectively, as confirmed by RR, which allows the quantification of the increased efficacy of a drug in the presence of a chemosensitizing agent (Table 3). Instead, less potentiation was observed in Mz-ChA-1 cell line, where sorafenib cytotoxicity was increased by 6% and 3% at 1 µg/mL and by 15% and 20% at 10 µg/mL in combination with verapamil and MK571, respectively (Figure 3B). Altogether, in combination with verapamil and MK571, a 1.5- and 1.7-fold reduction of sorafenib IC_50_ was achieved (Table 3). Finally, in Bx-PC3 cells, sorafenib cytotoxicity was potentiated by about 3% and 10% at 1 µg/mL, and by about 27% and 47% at 10 µg/mL in combination with verapamil and MK571 (Figure 3C): accordingly, approximately a 2- and 3.9-fold lower sorafenib IC_50_ was achieved, respectively (Table 3). Analogously, the nontoxic concentration of 10 µg/mL of caryophyllane sesquiterpenes sensitized cancer cells to sorafenib treatment. In particular, in HepG2 cells, the combination with β-caryophyllene and β-caryophyllene oxide determined a 5% and 20% increase of sorafenib cytotoxicity at the concentration of 1 µg/mL, despite a null effect of the anticancer drug alone. The inhibition of cell viability was even more pronounced at 10 µg/mL; indeed, sorafenib cytotoxicity was increased by approximately 33% and 44% with β-caryophyllene and β-caryophyllene oxide, respectively (Figure 3D). Overall, sorafenib IC_50_ was reduced by approximately 2.9- and 6-fold in the presence of β-caryophyllene and β-caryophyllene oxide (Table 3).

In Mz-ChA-1, a less reduction was highlighted with respect to HepG2 cells. Indeed, the combination with β-caryophyllene and β-caryophyllene oxide determined a lower increase of sorafenib cytotoxicity at the concentration of 10 µg/mL, being equal to 19% and 14%, respectively. Moreover, a potentiation of 17% and 13% was highlighted in combination with sorafenib at the concentration of 50 µg/mL (Figure 3E). IC_50_ of sorafenib was reduced by approximately 1.7-fold and 1.5-fold in the presence of β-caryophyllene and β-caryophyllene oxide, thus being the first compound to be slightly more effective (Table 3). Similarly, both sesquiterpenes potentiated sorafenib in Bx-PC3 cells; indeed, sorafenib cytotoxicity was increased by 56% and 64%, respectively (Figure 3F). Interestingly, almost a 20% potentiation was already found at the concentration of 1 µg/mL for both sesquiterpenes. Accordingly, the sorafenib IC_50_ was 5- and 9-fold lower in the presence of β-caryophyllene and β-caryophyllene oxide, respectively (Table 3).

Based on these results, the interaction nature between sorafenib and caryophyllane sesquiterpenes was characterized by applying the combination index method (CI). According to Di Giacomo et al. [26], a combination index (CI) value lower than 1 highlights a synergistic interaction, while an additive effect occurs when this value is equal to 1; conversely, if CI is higher than 1, the interaction is considered antagonistic.

Under our experimental conditions, the combination of sorafenib with β-caryophyllene produced CI values of 0.47, 0.74, and 0.46 in HepG2, Mz-ChA-1, and Bx-PC3 cells, respectively, while in association with β-caryophyllene oxide of 0.29, 0.76, and 0.26, respectively. When combined with verapamil and MK571, the following CI values were obtained: 0.33 and 0.20 in HepG2, 0.68 and 0.60 in Mz-ChA-1, and 0.51 and 0.27 in Bx-PC3 cells, respectively.

**Figure 4:**
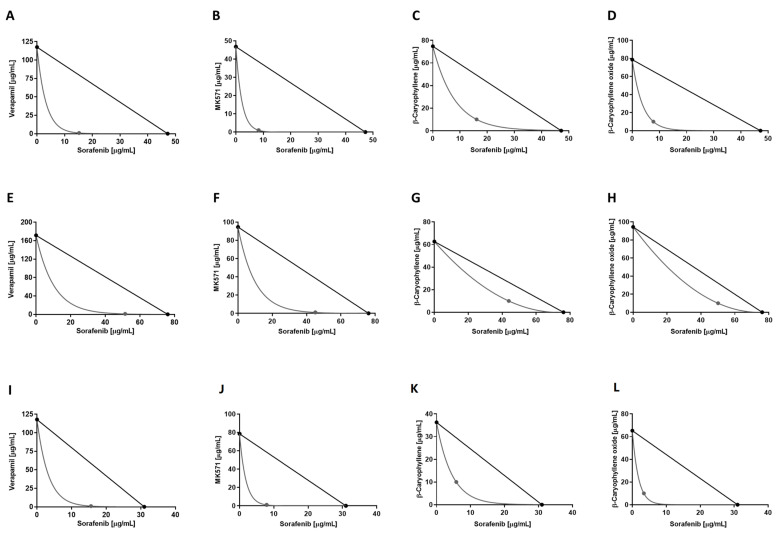
Isobolographic analysis of the cytotoxic effect of sorafenib in combination with verapamil and MK571 (1 μg/mL), and with β-caryophyllene and β-caryophyllene oxide (10 μg/mL) in HepG2 (**A**–**D**), Mz-ChA-1 (**E**–**H**), and Bx-PC3 (**I**–**L**) cells.

Overall, the interactions between sorafenib and caryophyllane sesquiterpenes appear to be always due to synergistic mechanisms. The isobologram analysis agreed with the CI values and highlighted synergistic effects of the caryophyllane sesquiterpenes with sorafenib in hepatic, biliary, and pancreatic cancer cells (Figure 4).

**Figure 5:**
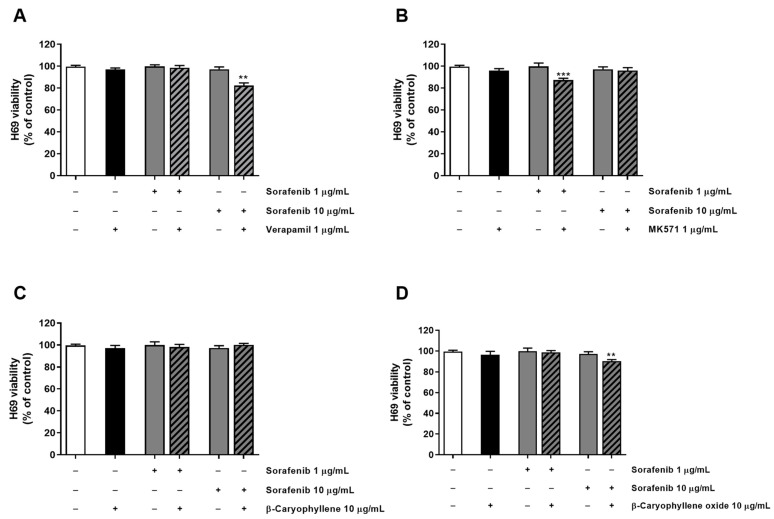
Cytotoxicity of sorafenib in combination with verapamil (**A**), MK571 (**B**), β-caryophyllene (**C**), and β-caryophyllene oxide (**D**) in noncancerous H69 cholangiocytes after 4 h exposure and 72 h recovery time. Data displayed as mean ± SE of at least three independent experiments (*n* = 3). ** *p* < 0.01 and *** *p* < 0.001 vs. sorafenib alone (Student’s *t*-test).

Tolerability of the combination of sorafenib and test substances was also investigated in H69 noncancerous cholangiocytes by applying the same exposure protocol used for the sorafenib chemosensitization. As expected, the combination of the anticancer drug with both verapamil and MK571 increased the cytotoxicity of 1 µg/mL and 10 µg/mL sorafenib, producing almost a 15% reduction of cell viability with respect to sorafenib alone (Figure 5). Conversely, caryophyllane sesquiterpenes (10 μg/mL) did not affect sorafenib cytotoxicity; even better, β-caryophyllene produced slight protective effects from the damage induced by sorafenib at the concentration of 10 μg/mL (Figure 5).

Present results confirm our previous evidence about the synergistic interaction of caryophyllane sesquiterpene and sorafenib not only in hepatic but also in biliary and pancreatic cells, which share a common embryological origin, along with protective effects in noncancerous cells.

### 3.3. Caryophyllane Sesquiterpenes Inhibit Pgp and MRP1/2 Transporter Activity in Human Hepato-Biliary-Pancreatic Cancer Cell Lines

The ability of β-caryophyllene and β-caryophyllene oxide to inhibit Pgp and MRP1/2 activity was investigated in HepG2, Mz-ChA-1, and Bx-PC3 cells by using a preincubation protocol to make sure the compounds were inside the cells when these were exposed to the substrates. Cells were pretreated with the test substances and positive controls for two hours; then, the fluorescent probes rhodamine 123 and calcein acetoxymethyl ester (substrates of Pgp and MRP1/2 respectively) were added. The potential inhibition of other ABC transporters was not investigated based on our previous work, in which a lower effect of caryophyllane sesquiterpenes on them was highlighted [20].

**Figure 6:**
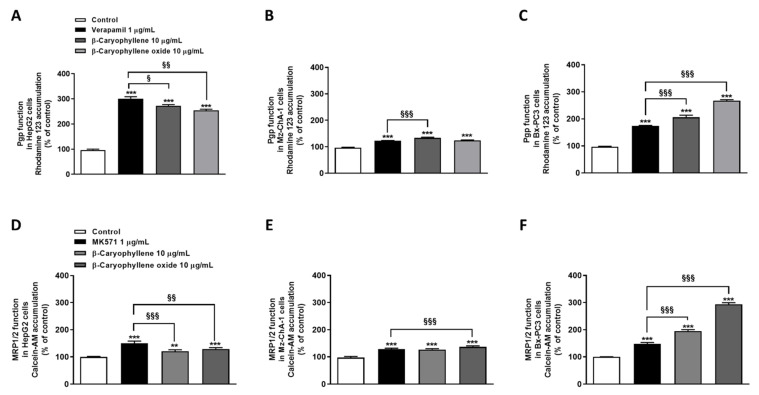
Effect of β-caryophyllene and β-caryophyllene oxide on the intracellular accumulation of rhodamine 123 and calcein acetoxymethyl ester (Calcein-AM) in HepG2 (**A**,**D**), Mz-ChA-1 (**B**,**E**), and Bx-PC3 (**C**,**F**) cells. ** *p* < 0.01 and *** *p* < 0.001 (*t*-Student’s test) vs. control. ^§^ *p* < 0.05, ^§§^ *p* < 0.01, and ^§§§^ *p* < 0.001 (*t*-Student’s test) vs. positive controls (i.e., verapamil or MK571).

Under our experimental conditions, both sesquiterpenes were able to impair Pgp functional activity. Specifically, β-caryophyllene raised rhodamine 123 accumulation by approximately 2.7-, 1.3-, and 2.0-fold in HepG2, Mz-ChA-1, and Bx-PC3 cells, while β-caryophyllene oxide by 2.5-, 1.2-, and 2.7-fold, respectively. The positive control verapamil exhibited the highest effect in HepG2 cells, inducing a 3-fold increase of rhodamine 123 accumulation; conversely, its effect in Mz-ChA-1 and Bx-PC3 cells was lower than that of caryophyllane sesquiterpenes, as the rhodamine 123 accumulation increased by only 1.2- and 1.7-fold (Figure 6A–C).

A significant reduction of calcein-AM efflux, due to the inhibition of MRP1/2 activity, was observed after cell treatment with the test substances, especially with β-caryophyllene oxide. Specifically, β-caryophyllene oxide determined an increase of calcein-AM accumulation by approximately 1.3-, 1.4-, and 3.0-fold in HepG2, Mz-ChA-1, and Bx-PC3 cells, respectively, despite a lower effect (i.e., 1.2-, 1.3-, and 1.9-fold increase of the fluorescent substrate accumulation in HepG2, Mz-ChA-1, and Bx-PC3 cells, respectively) by β-caryophyllene. Interestingly, while in HepG2 and Mz-ChA-1 cells a higher or comparable effect with respect to caryophyllane sesquiterpenes was highlighted for MK571, in Bx-PC3 cells a lower inhibition of MRP1/2 activity was observed: calcein-AM accumulation raised by only 1.5-fold (Figure 6D–F).

Overall, β-caryophyllene oxide was shown to be the most effective compound in inhibiting Pgp and MRP1/2 activity, especially in Bx-PC3 cells, although a remarkable effect was also induced by β-caryophyllene. Based on these results and considering the high potentiation of sorafenib cytotoxicity in combination with both sesquiterpenes in Bx-PC3 cells, this cell line was selected to further investigate the underlying mechanisms of sorafenib chemosensitization by caryophyllane sesquiterpenes.

### 3.4. Modulation of MDR1, MRP1, and MRP2 Expression Is Involved in Sorafenib Chemosensitization by Caryophyllane Sesquiterpenes

**Figure 7:**
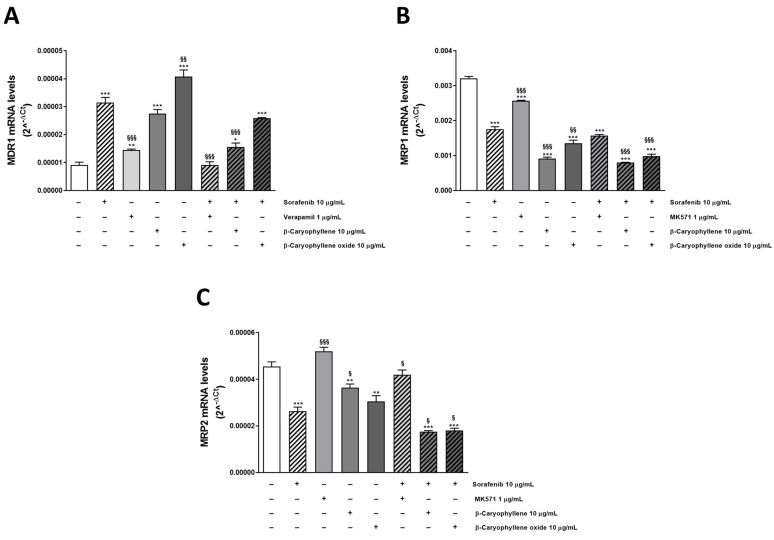
Relative mRNA expression of MDR1 (**A**), MRP1 (**B**), and MRP2 (**C**) in Bx-PC3 cells after treatment with sorafenib, verapamil, MK571, β-caryophyllene, β-caryophyllene oxide, and their combination with the anticancer drug after 4 h exposure and 72 h recovery time. * *p* < 0.05, ** *p* < 0.01 and *** *p* < 0.001 (one-way ANOVA followed by Dunnett’s multiple comparison post test) vs. control. ^§^ *p* < 0.05, ^§§^ *p* < 0.01, and ^§§§^ *p* < 0.001 (one-way ANOVA followed by Dunnett’s multiple comparison post test) vs. sorafenib alone.

After ascertaining the direct inhibition of transporters by tested substances, we wondered if the chemosensitizing effect of caryophyllane sesquiterpenes could be related to a modulation of MDR1, MRP1, and MRP2 expression too. To this end, we performed mechanistic studies in Bx-PC3 cells, which was the most sensitive to the combined treatments. In fact, RT-qPCR analysis highlighted the ability of β-caryophyllene and β-caryophyllene oxide to affect the ABC transporter expression at gene level. All the substances alone upregulated MDR1 gene, with sorafenib and β-caryophyllene oxide being the most effective, followed by β-caryophyllene and verapamil (increase of MDR1 expression by 3.6-, 4.7-, 2.7-, and 1.7-fold with respect to control). Conversely, the combinations of sorafenib with verapamil, β-caryophyllene, and β-caryophyllene oxide determined a 3.7-, 1.9-, and 1.3-fold downregulation of MDR1 with respect to the anticancer drug alone (Figure 7A).

Surprisingly, the test substances induced a downregulation of MRP1 and MRP2; sorafenib decreased their expression by almost 2-fold, achieving a 4- and 3-fold reduction in combination with β-caryophyllene and β-caryophyllene oxide, respectively. Similarly, the positive control MK571 affected MRPs expression, although to a lower extent, with respect to caryophyllane sesquiterpenes (Figure 7B,C).

Overall, RT-qPCR analysis showed that MRP1 was the most expressed transporter in our cellular model, its levels being 352- and 71-fold higher than those of MDR1 and MRP2, respectively. Our findings are consistent with the potentiation of sorafenib cytotoxicity observed in the combination studies; indeed, the Pgp inhibitor verapamil was the less effective in increasing sorafenib efficacy.

In order to confirm the involvement of Pgp, MRP1, and MRP2 transporter modulation in the chemosensitizing effect of caryophyllane sesquiterpenes and to verify their presence in the tested cells, immunofluorescence analysis has been performed as well.

**Figure 8:**
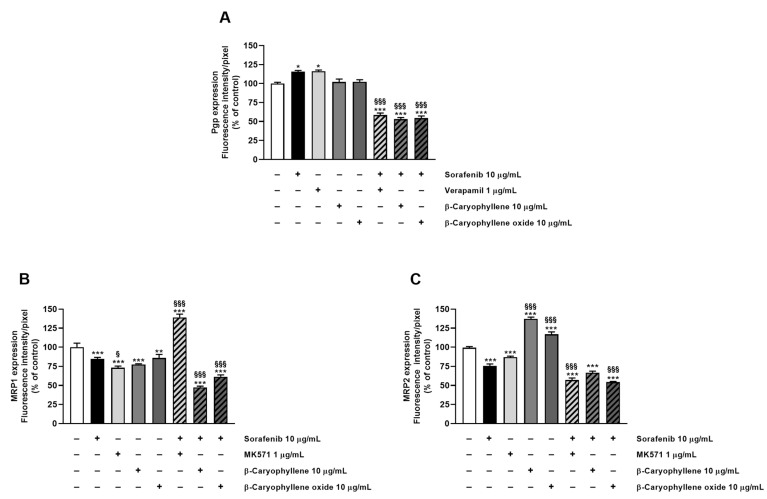
Densitometric analysis of (**A**) Pgp, (**B**) MRP1, and (**C**) MRP2 immunofluorescence carried out by Gen5™ Microplate Reader and Imager Software 3.11. * *p* < 0.05, ** *p* < 0.01, and *** *p* <0.001 (ANOVA + Dunnett’s multiple comparison post test) vs. control. ^§^ *p* < 0.05, ^§§§^ *p* < 0.001 (ANOVA + Dunnett’s multiple comparison post test) vs. sorafenib.

**Figure 9:**
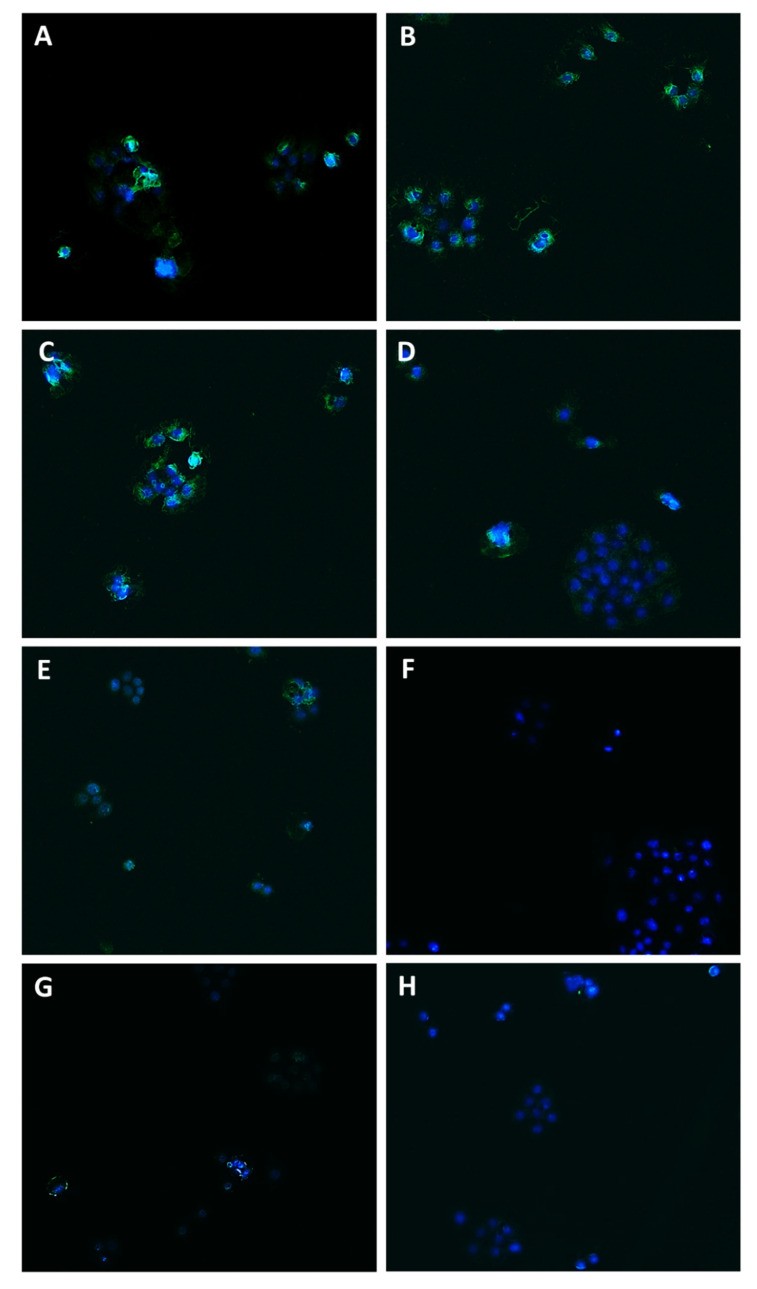
Modulation of Pgp transporter expression in Bx-PC3 cells detected after 4 h exposure and 72 h recovery time. Representative images obtained at immunofluorescence analysis. Original magnification: 10×. (**A**) Control; (**B**) sorafenib 10 µg/mL; (**C**) verapamil 1 µg/mL; (**D**) verapamil 1 µg/mL + sorafenib 10 µg/mL; (**E**) β-caryophyllene 10 µg/mL; (**F**) β-caryophyllene 10 µg/mL + sorafenib 10 µg/mL; (**G**) β-caryophyllene oxide 10 µg/mL; (**H**) β-caryophyllene oxide 10 µg/mL + sorafenib 10 µg/mL.

**Figure 10:**
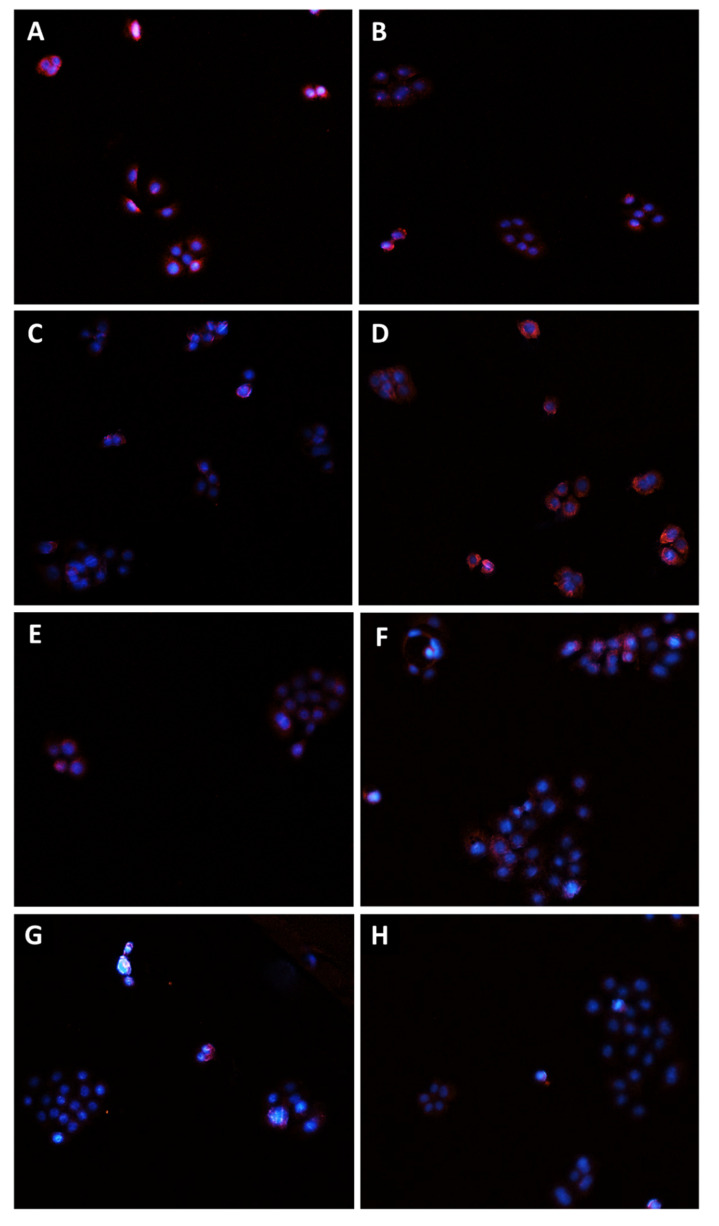
Modulation of MRP1 transporter expression in Bx-PC3 cells detected after 4 h exposure and 72 h recovery time. Representative images obtained at immunofluorescence analysis. Original magnification: 10×. (**A**) Control; (**B**) sorafenib 10 µg/mL; (**C**) MK571 1 µg/mL; (**D**) MK571 1 µg/mL + sorafenib 10 µg/mL; (**E**) β-caryophyllene 10 µg/mL; (**F**) β-caryophyllene 10 µg/mL + sorafenib 10 µg/mL; (**G**) β-caryophyllene oxide 10 µg/mL; (**H**) β-caryophyllene oxide 10 µg/mL + sorafenib 10 µg/mL.

**Figure 11:**
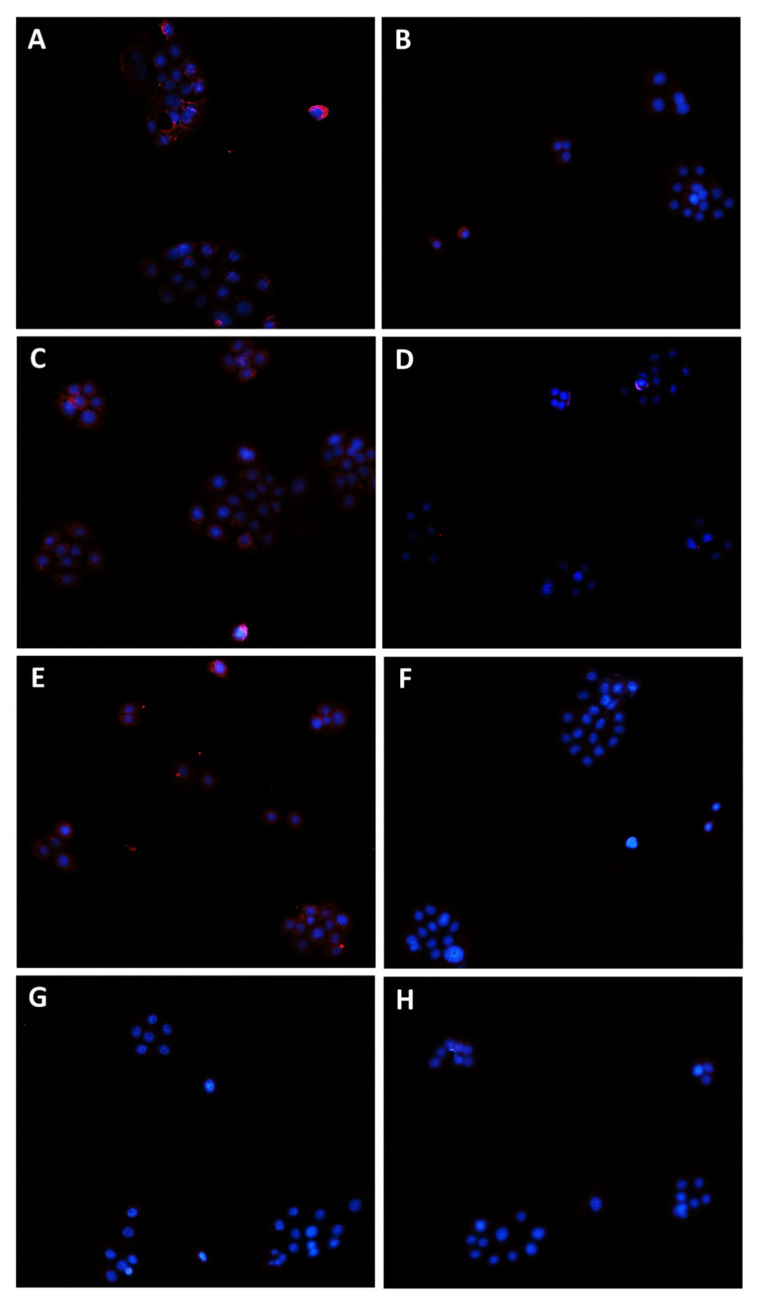
Modulation of MRP2 transporter expression in Bx-PC3 cells detected after 4 h exposure and 72 h recovery time. Representative images obtained at immunofluorescence analysis. Original magnification: 10×. (**A**) Control; (**B**) sorafenib 10 µg/mL; (**C**) MK571 1 µg/mL; (**D**) MK571 1 µg/mL + sorafenib 10 µg/mL; (**E**) β-caryophyllene 10 µg/mL; (**F**) β-caryophyllene 10 µg/mL + sorafenib 10 µg/mL; (**G**) β-caryophyllene oxide 10 µg/mL; (**H**) β-caryophyllene oxide 10 µg/mL + sorafenib 10 µg/mL.

Staining of Bx-PC3 cells with specific antibodies corroborated the RT-qPCR findings, confirming the presence of Pgp, MRP1, and MRP2 transporters on cell surface. ABC pumps appeared differently modulated by the treatments (Figure 8, Figure 9, Figure 10 and Figure 11): a slight increase of Pgp expression was observed after treatment with sorafenib and verapamil alone, while β-caryophyllene and β-caryophyllene oxide did not affect it. Conversely, the combination of sorafenib with verapamil, β-caryophyllene, and β-caryophyllene oxide determined a marked reduction of Pgp expression by approximately 1.7-, 1.9-, and 1.8-fold compared to control, respectively (Figure 8A and Figure 9).

According to RT-qPCR results, MRP1 expression was slightly lowered by the treatments with sorafenib (1.2-fold compared to control) and caryophyllane sesquiterpenes (1.4-fold compared to control) alone, achieving a 1.8- and 1.5-fold reduction with the combination of sorafenib with β-caryophyllene and β-caryophyllene oxide with respect to sorafenib. Conversely, when assessed with the positive control MK571, a 1.4-fold increase of MRP1 expression on cell surface with respect to the control was found (Figure 8B and Figure 10).

At last, MRP2 transporter expression in Bx-PC3 cells was found to be significantly lowered by sorafenib, MK571, and their combination, while β-caryophyllene and β-caryophyllene oxide alone determined its 1.4- and 1.2-fold increase (Figure 8C and Figure 11). Surprisingly, the combined treatment of sorafenib with β-caryophyllene and β-caryophyllene oxide affected the MRP2 expression, inducing a 1.3- and 1.6-fold reduction with respect to sorafenib (Figure 8C and Figure 11).

Altogether, our results suggest that the lowered expression of ABC transporters on the surface of Bx-PC3 cells could be due to a downregulation of gene expression induced by caryophyllane sesquiterpenes and could support our hypothesis on the involvement of Pgp, MRP1, and MRP2 modulation in the chemosensitizing effects of β-caryophyllene and β-caryophyllene oxide.

### 3.5. Modulation of STAT3 Activation and Cell Migration in Sorafenib Chemosensitization by Caryophyllane Sesquiterpenes

Taking into account the results obtained in the combination experiments that showed a potentiation of sorafenib cytotoxicity by caryophyllane sesquiterpenes, especially β-caryophyllene oxide, and the recognized involvement of ABC-transporters and STAT3 signaling in the progression and chemoresistance of hepato-biliary-pancreatic cancers [27,28,29,30,31,32,33], a possible modulation by treatments of this cascade in Bx-PC3, associated with the observed changes in MDR1 and MRPs pumps, was also evaluated (Figure 11). Activation of STAT3 by phosphorylation at tyrosine 705 (phospho(Tyr705)-STAT3), which is known to play an important role in the control of cell survival, proliferation, and apoptosis [34], has been measured.

**Figure 12:**
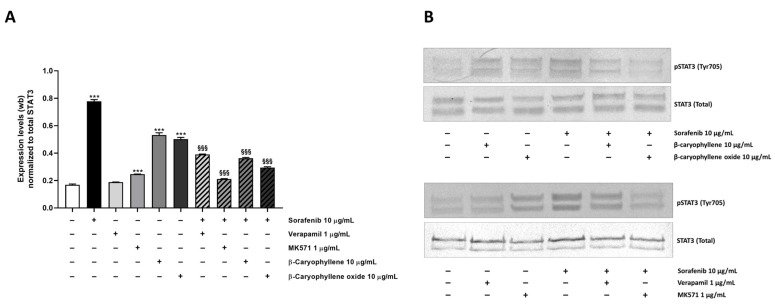
Effect of sorafenib, caryophyllane sesquiterpenes, verapamil, MK571, and their combination on the expression levels of phosphorylated STAT3 on tyrosine 705 residue in Bx-PC3 cells after 4 h exposure and 72 h recovery time. (**A**) Densitometric bar graph analysis (data expressed as mean ± standard error obtained from at least two independent experiments). (**B**) Representative Western blotting images, displaying phospho(Tyr705) STAT3 and total STAT3 (protein loading control). *** *p* < 0.001 (one-way ANOVA followed by Dunnett’s multiple comparison post-test) vs. control. ^§§§^ *p* < 0.001 (one-way ANOVA followed by Dunnett’s multiple comparison post-test) vs. sorafenib.

Under our experimental conditions, a 4.6-fold increase of STAT3 phosphorylation at tyrosine 705 residue was induced by sorafenib (Figure 12A,B). Similarly, β-caryophyllene and β-caryophyllene oxide raised STAT3 phosphorylation by approximately 3-fold, but to a lower extent than the anticancer drug. The positive controls verapamil and MK571 induced a 1.1- and 1.5-fold increase of STAT3 phosphorylation too.

Combination of caryophyllane sesquiterpenes and sorafenib led to a significant downregulation of phospho(Tyr705)-STAT3 with respect to the anticancer drug alone (Figure 12A,B). The inhibitory effect was especially marked in combination with β-caryophyllene oxide, being phospho(Tyr705)-STAT3 expression reduced by 2.6-fold respect to sorafenib; a lower but significant 2.1-fold reduction also occurred in combination with β-caryophyllene. The combination of sorafenib with the positive control MK571 resulted the most effective treatment, being able to reduce STAT3 phosphorylation by 3.7-fold; conversely, a lower reduction occurred in the presence of verapamil (Figure 12 and Figures S1–S4).

**Figure 13:**
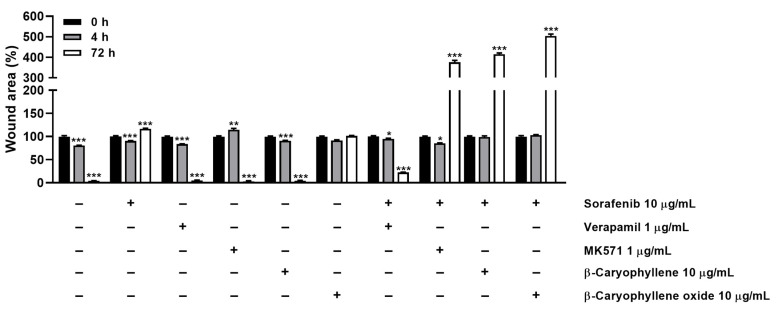
Quantification of Bx-PC3 cells migration rate analyzed by ImageJ at 0 h, 4 h, and 72 h (*n* = 3) after treatment with sorafenib (10 µg/mL), β-caryophyllene (10 µg/mL), β-caryophyllene oxide (10 µg/mL), verapamil (1 µg/mL), and MK571 (1 µg/mL) alone or in combination with the anticancer drug. Results are reported as mean ± SE of wound area percentage of at least two independent experiments (*n* = 2). * *p* < 0.05, ** *p* < 0.01, and *** *p* < 0.001 (one-way ANOVA followed by Dunnett’s multiple comparison post-test) vs. wound area percentage at 0 h.

**Figure 14:**
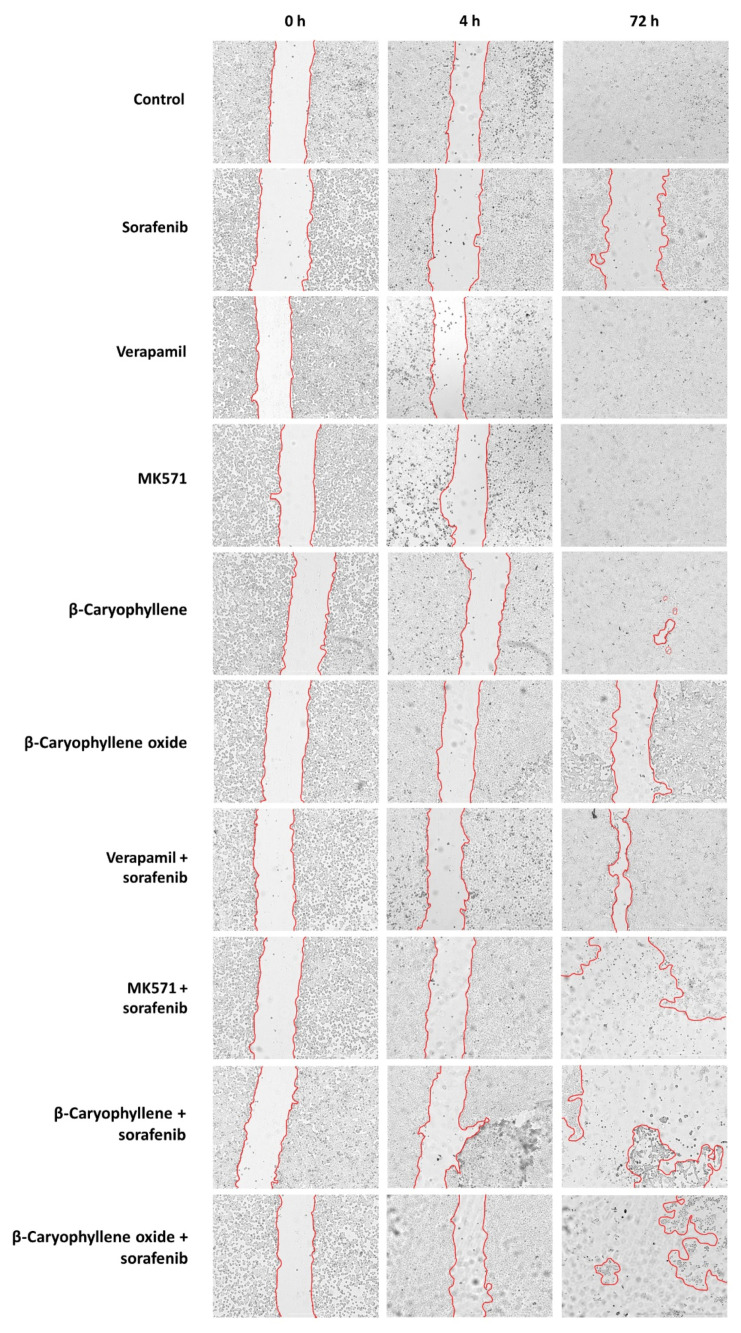
Wound images of Bx-PC3 cells at 0 h, 4 h, and 72 h after treatment with sorafenib (10 µg/mL), verapamil (1 µg/mL), MK571 (1 µg/mL), β-caryophyllene (10 µg/mL), and β-caryophyllene oxide (10 µg/mL), alone or in combination with the anticancer drug. Scale bar: 1000 μm.

In order to better disclose the involvement of STAT3 and ABC transporter modulation by caryophyllane sesquiterpenes in Bx-PC3 pancreatic cancer cell proliferation and progression, the cell migration in the wound healing assay was studied (Figure 13 and Figure 14). Under our experimental conditions, the treatment of Bx-PC3 cells with sorafenib determined a slight reduction of wound area after 4 h, while a 1.2-fold increase was observed at 72 h. Verapamil, MK571, and β-caryophyllene showed a similar trend with respect to the control; indeed, after 72 h of treatment, an almost complete closure of wound area was highlighted. Conversely, β-caryophyllene oxide blocked cell migration, the wound area after 72 h being similar to that of the zero time. Interestingly, the combination of sorafenib with test substances showed a decrease of wound healing. Particularly, MK571, β-caryophyllene, and β-caryophyllene oxide were the most effective, increasing the wound area by approximately 3.7-, 4.1-, and 5-fold, respectively; conversely, the combination with verapamil lowered the anti-migration effect of sorafenib.

The present results are in agreement with the modulation of STAT3 activation observed for the combination of sorafenib with MK571, β-caryophyllene, and β-caryophyllene oxide in the Western blotting analysis and suggest that sorafenib chemosensitization by caryophyllane sesquiterpenes increases both cytotoxicity and antimetastatic abilities of the anticancer drug in Bx-PC3 cells.

## 4. Discussion and Conclusions

Chemoresistance, namely the ability of cancer cells to escape from antitumor drug-induced damage, represents the major obstacle to the effective treatment of several cancers, leading to the failure of chemotherapy and consequent tumor relapse [35]. It can occur as a result of both intrinsic and extrinsic mechanisms: intrinsic resistance involves factors already present in cancer cells or tissues and are activated in response to anticancer treatment to counteract chemotherapeutic effects, while extrinsic or acquired drug resistance is developed during treatment in sensitive cancer cells and leads to adaptive responses, which finally impair chemotherapy efficacy [36]. Different mechanisms are involved in this phenomenon, such as activation of survival cascades, escaping growth suppressors, and inactivation of cell death; moreover, complex processes can directly affect the anticancer drugs, through metabolic inactivation or elimination mechanisms, thus lowering their therapeutic efficacy [35].

Several cancers have been found to be characterized by an overexpression of ATP-binding cassette (ABC) transporters, which are membrane proteins responsible for drug efflux from cells, whereby their overexpression in cancer cells can reduce the anticancer drug bioavailability and favor multidrug resistance (MDR) development [37]. This represents a common drawback for many anticancer drugs such as tyrosine kinase inhibitors, anthracyclines, and taxanes [38,39,40,41].

Notably, the upregulation of ABC pumps may be present in tumor tissues prior to treatment (intrinsic resistance) or may develop after the drug treatment of tumors that are initially responsive (acquired resistance) [42,43]. Recently, a biological role in tumor growth and progression has been highlighted for ABC transporters [44]. Indeed, an increase of their expression and activity has been related to tumor progression and metastatic potential and, consequently, to poorer clinical prognosis [45].

Hepatocellular carcinoma (HCC), cholangiocarcinoma (CCA) and ductal adenocarcinoma (PDAC) are examples of tumors in which ABC transporters play this dual role; in particular, an upregulation of MDR1 and MRP1 is correlated with a more aggressive phenotype of these cancers, characterized by reduced differentiation, larger tumors, and the presence of microvascular invasion, while MRP2 expression has been mainly associated with poorer response to antitumor drugs [20,27,28,29]. Therefore, targeting ABC transporter function and expression could represent an important strategy to fight cancer progression and to overcome chemoresistance, thus increasing the anticancer drug effectiveness at low doses and reducing the chemotherapy side effects.

In line with this evidence, in the present study, we found that the natural sesquiterpenes β-caryophyllene and β-caryophyllene oxide were able to synergistically potentiate the anticancer effects of sorafenib in hepatoblastoma (HepG2), extrahepatic cholangiocarcinoma (Mz-ChA-1), and pancreas adenocarcinoma (Bx-PC3) cells, with the effect being the most pronounced in the latter cell line.

Present findings agree with our previous evidence in liver and cholangiocarcinoma cells; in particular, we found that both sesquiterpenes were able to potentiate doxorubicin efficacy in HepG2 cells after both a long-term exposure of 24 h and under a metronomic schedule, with β-caryophyllene oxide being the most effective chemosensitizing compound [13]. Under the same treatment protocols, β-caryophyllene increased doxorubicin cytotoxicity in cholangiocarcinoma Mz-ChA-1 cells [21].

β-Caryophyllene oxide also produced synergistic effects towards doxorubicin and other anticancer drugs in liver, breast, leukemic, and colon cancer cells, especially in resistant cells [13,15,19,46,47]; furthermore, it exhibited the ability to restore liver cancer sensitivity to sorafenib both in vitro and in vivo [20]. Based on this evidence, the combination of caryophyllane sesquiterpenes and anticancer drugs appears to be an interesting strategy to increase the drug potency and to reduce their toxicity; however, further in vivo studies are required in support of a future pharmacological development.

Interestingly, under our experimental conditions, we highlighted the ability of β-caryophyllene to reduce sorafenib toxicity in noncancerous H69 cholangiocytes, despite a null effect of β-caryophyllene oxide and an increased cytotoxicity by the standard ABC-transporter inhibitors verapamil and MK571. Accordingly, previous studies have shown a protective effect of β-caryophyllene towards the damage induced by the anticancer drug doxorubicin in both cardiomyocytes and cholangiocytes [21,48]. Moreover, the safety of the combined treatment between β-caryophyllene and doxorubicin was also confirmed in vivo after a single administration in rats [21]. Regarding β-caryophyllene oxide, although its tolerability in combination with anticancer drugs has never been tested, our previous in vivo study highlighted the safety of this compound up to the concentration of 100 mg/kg [20]. These results suggest that the combination with caryophyllane sesquiterpenes could be a novel strategy to achieve both an increased chemotherapy efficacy and drug tolerability. Notably, both β-caryophyllene and β-caryophyllene oxide are approved as flavorings by the European Food Safety Authority and are considered safe up to values of 222 and 109 mg/kg/die, respectively [15].

Our results also highlighted that the increased sensitivity of cancer cells to sorafenib treatment was due to the inhibition of Pgp and MRP1/2 activity, especially in Bx-PC3 cells, in which β-caryophyllene oxide exerted a higher effect with respect to β-caryophyllene. Several studies have demonstrated that ABC transporters play a central role in sorafenib resistance [39]. Indeed, different pumps, including MDR1, MRP1, MRP2, MRP3, and BCRP, have been reported to export the anticancer drug from cells, and thus are associated with sorafenib resistance [49,50,51]. In this landscape, we decide to investigate the effect of caryophyllane sesquiterpenes towards MDR1, MRP1, and MRP2 pumps based on the results obtained in our previous work, which highlighted a lower effect of these compounds on MRP3 transporter [20].

Considering the high potentiation of sorafenib cytotoxicity in combination with both substances in Bx-PC3 cells, this cell line was selected to further investigate the underlying mechanism of sorafenib chemosensitization. Indeed, although a direct inhibition of ABC pumps was exerted by tested compounds, we hypothesized that the chemosensitizing effect of caryophyllane sesquiterpenes could be related to the modulation of MDR1, MRP1, and MRP2 expression at both gene and protein levels, also in consideration of the treatment protocol applied in the present study.

Under our experimental conditions, sorafenib increased the MDR1 expression, while downregulating both MRP1 and MRP2. Intriguingly, the combination of the anticancer drug with both caryophyllane sesquiterpenes reduced the expression of all transporters with respect to sorafenib alone. Therefore, present results support our hypothesis about the involvement of a Pgp, MRP1, and MRP2 modulation in the chemosensitizing effect of β-caryophyllene and β-caryophyllene oxide. Moreover, it is noteworthy that a higher expression of MRP1 with respect to both MRP2 and MDR1 was highlighted at RT-qPCR analysis, thus suggesting the greater involvement of MRP1 in the chemosensitizing effects of the tested compounds.

MRP1 is a transporter of neutral and anionic hydrophobic compounds and products of phase II drug metabolism, including glucuronide- and glutathione-conjugates [37]. It exerts physiological and protective functions, being able to pump out both endogenous agents and xenobiotics; aside from its role in anticancer drug efflux, this transporter could contribute to cancer cell chemoresistance by regulating the GSH/GSSG ratio inside them [37]. Indeed, high levels of MRP1 have been related to an increased efflux of GSSG, as a mechanism for cellular redox homeostasis maintenance, so becoming an advantageous to cancer cells [52]. Consequently, the reduction of MRP1 expression induced by the combined treatment could be exploited to increase sorafenib concentration inside the cancer cells and to reduce their viability, owing to an imbalance of oxidative state. Moreover, MRP2 is responsible for the transport of toxic complexes and regulates the excretion of bilirubin and bile secretion in the body, thereby conferring protection to body against toxins [37]. A downregulation of MRP2 expression has been associated with liver disfunctions and inflammation [37,53,54,55]. Moreover, an increased MRP2 mRNA expression has been found to be associated with chemoresistance of pancreatic cancer to gemcitabine plus cisplatin [56]. Similarly, reducing MRP1 expression in pancreatic cancer cell lines resulted in enhanced gemcitabine sensitivity [57], thus suggesting that MRPs play a pivotal role in the efflux control of pancreatic cells.

Under our experimental conditions, we found that MRP1 was the most expressed transporter in Px-PC3 pancreatic cancer cells, with lower levels of both MRP2 and MDR1; moreover, MRP1 and MRP2 were downregulated by sorafenib, according to previous studies [58]. In particular, tyrosine kinase inhibitors usually lowered the expression of these transporters in early treatment phases, thus being able to restore the sensitivity to conventional anti-neoplastic drugs (e.g., doxorubicin) when used in combination [45,59]. However, an upregulation of both MRP1 and MRP2, and MDR1, MRP3, and BCRP transporters has been also reported after prolonged treatment, thus contributing to sorafenib resistance [49].

Finally, the involvement of STAT3 signaling–a critical hub in the transcriptional regulation of genes involved in survival, progression, and invasion of cancer cells such as those of hepato-biliary-pancreatic system [20,60,61]—in ABC transporter modulation was investigated, based on evidence in the literature showing its involvement in regulating the transcription of MDR1 and MRP1 transporters [62].

Under our experimental conditions, sorafenib treatment determined a high increase of STAT3 phosphorylation at tyrosine 705 residue. Similarly, β-caryophyllene and β-caryophyllene oxide were able to raise STAT3 phosphorylation, although to a lower extent than the anticancer drug. Conversely, the combination of caryophyllane sesquiterpenes and sorafenib determined a significant downregulation of phospho(Tyr705)-STAT3 compared to the anticancer drug alone. This inhibitory effect was higher in combination with β-caryophyllene oxide, although a lower reduction occurred in combination with β-caryophyllene too. The marked inhibition of STAT3 exerted by β-caryophyllene oxide could partly explain the higher sorafenib chemosensitization in Bx-PC3 cells with respect to β-caryophyllene. Indeed, several studies have shown that enhancing the STAT3 activation promotes pancreatic cancer malignance and is strongly associated with poor prognosis [30], thus suggesting that targeting STAT3 could potentially be exploited as a promising strategy to fight pancreatic cancer.

Our results, although in line with the recognized mechanisms of chemoresistance to sorafenib [39,63,64], disagree with a previous study highlighting a reduction of STAT3 phosphorylation at tyrosine 705 residue following sorafenib treatment in Bx-PC3 cells [65]. However, it should be outlined that a different treatment protocol was used, thus partially explaining the difference observed. In any case, the literature reported controversial results in relation to the role of sorafenib in STAT3 regulation [30,64,65]; therefore, further mechanistic studies both in vitro and in vivo cancer models are expected.

Regarding the possible link between STAT3 cascade and ABC transporters, our results highlight a positive correlation only with MDR1 expression, while MRP1 and MRP2 seem not to be under the control of STAT3, at least in our cellular model, although more specific studies are required in confirmation.

In order to clarify if the modulation of STAT3 and ABC transporters by caryophyllane sesquiterpenes in combination with sorafenib could control proliferation and progression of Bx-PC3 pancreatic cancer cells, the cell migration has been evaluated too. Indeed, STAT3 is known to regulate the expression of metastasizing factors in several malignancies, including pancreatic cancer, thus promoting proliferation, invasion, and metastasis processes [66,67,68]. On the other hand, recent evidence has highlighted the involvement of ABC transporters in the regulation of cancer cell proliferation, migration, and metastasis, although the true mechanisms are not fully understood [69].

Interestingly, under our experimental conditions, the combined treatment of sorafenib with β-caryophyllene, β-caryophyllene oxide, and MK571 markedly inhibited cell migration and induced cytotoxicity, in agreement with STAT3 phosphorylation results. Moreover, a similar behavior of caryophyllane sesquiterpenes and MK571 in sorafenib sensitization was highlighted, likely by involving MRPs transporters and suggesting a peculiar role of the STAT3/ABC transporter axis in the regulation of pancreatic cancer cell death.

**Figure 15:**
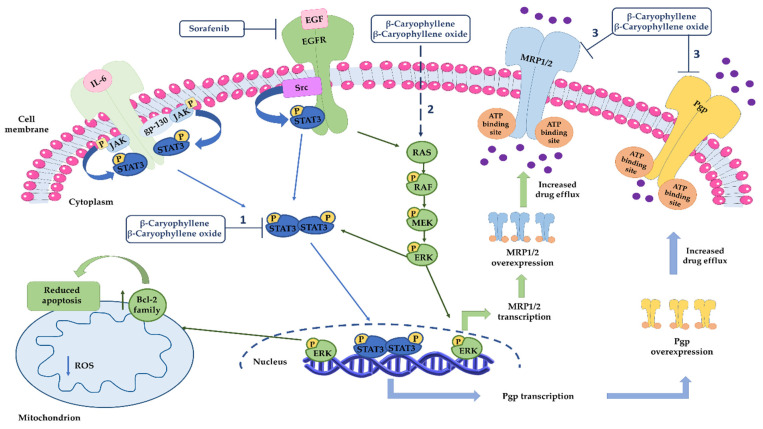
Mechanisms involved in sorafenib chemosensitization by caryophyllane sesquiterpenes. (1) β-Caryophyllene and β-caryophyllene oxide modulate Pgp expression through the inhibition of STAT3 signaling, which is involved in MDR1 gene overexpression in different cancer cells. (2) Caryophyllane sesquiterpenes can affect MRP1 and MRP2 expression likely by MAPK/ERK pathway, although the exact mechanism deserves further investigation. (3) β-Caryophyllene and β-caryophyllene oxide can directly inhibit Pgp and MRP1/2 pumps.

Based on the obtained results, our mechanistic hypothesis about sorafenib chemosensitization by caryophyllane sesquiterpenes is that they are able not only to potentiate sorafenib efficacy but also to counteract the activation of resistance mechanisms (Figure 15). In particular, sorafenib, being a multikinase inhibitor, can block the RAS/RAF/ERK cascade, leading to the inactivation of different transcription factors, among which are ABC transporters, and apoptosis; at mitochondrial level, the apoptotic cell death is also induced [70]. At the same time, it can activate both primary and acquired mechanisms of chemoresistance, including STAT3, which in turn regulates the expression of ABC transporter genes, such as mdr1. Caryophyllane sesquiterpenes can modulate Pgp expression induced by sorafenib, by inhibiting STAT3 activation, thus the mdr1 gene transcription. Moreover, they can impair the MRP1 and MRP2 expression, likely by affecting MAPK/ERK pathway, although the exact mechanism deserves further investigation. We postulate this hypothesis based on the known role of MAPK/ERK pathway in the transcription regulation of MRPs transporters [71]. Finally, β-caryophyllene and β-caryophyllene oxide can directly inhibit Pgp and MRP1/2 pumps, likely interacting with the hydrophobic space next to the nucleotide binding domain of ABC transporters [13]. Altogether our results provide novel insights in the mechanisms of sorafenib resistance in pancreatic cancers and strengthen the interest in exploiting caryophyllane sesquiterpenes as a novel cancer chemosensitization strategy.

## Figures and Tables

**Table 1:** List of primers, general conditions, and validation parameters used to perform RT-qPCR.

Gene	Brand	Primer (5′ → 3′)		Annealing (°C)	Efficiency (%)	R^2^
MDR1	Bio-Rad	Forward	N/A (Cod. qHsaCED0056970)	60	99.0	0.999
		Reverse				
MRP1	Bio-Rad	Forward	N/A (Cod. qHsaCID0016624)	60	98.0	0.998
		Reverse				
MRP2	Bio-Rad	Forward	N/A (Cod. qHsaCID0008411)	60	99.0	0.999
		Reverse				
GAPDH	Bio-Rad	Forward	N/A (Cod. qHsaCED0038674)	60	97.0	0.999
		Reverse				

**Table 2:** IC_50_ values of sorafenib, β-caryophyllene, β-caryophyllene oxide, and the positive controls verapamil and MK571 alone in hepatoblastoma (HepG2), extrahepatic cholangiocarcinoma (Mz-ChA-1), pancreas adenocarcinoma (Bx-PC3) cells, and noncancerous cholangiocytes (H69). Data displayed as mean ± SE of at least three independent experiments (*n* = 3).

Compound	IC_50_ [µg/mL] (CL)			
	HepG2	Mz-ChA-1	Bx-PC3	H69
Sorafenib	47.2 (43.7–51.0)	76.1 (72.2–80.2)	31.0 (28.4–33.7)	50.3 (45.0–55.58)
β-Caryophyllene	74.8 (71.6–78.2) ***	62.7 (60.3–65.2) **	36.3 (33.6–39.1)	67.0 (62.6–71.7) *
β-Caryophyllene oxide	78.7 (76.8–80.5) ***	94.5 (90.1–99.1) **	65.3 (62.1–68.7) ***	99.7 (93.1–106.9) ***
Verapamil	117.5 (107.6–128.3) ***	171.5 (159.3–188.9) ***	117.9 (112.3–124.3) ***	87.9 (78.7–98.71) **
MK571	46.8 (43.8–50.1)	94.6 (89.3–100.4) **	78.5 (73.0–84.3) ***	32.6 (27.9–37.8) *

CL, confidence limits. * *p* < 0.05, ** *p* < 0.01 and *** *p* < 0.001, significantly different than sorafenib IC_50_ value within the same cell line (Student’s *t*-test).

**Table 3:** IC_50_ values of sorafenib and its combination with β-caryophyllene, β-caryophyllene oxide, verapamil, and MK571 under the scheduled protocol.

Compound	IC_50_ [µg/mL] (CL) *RR* ^a^		
	HepG2	CCA	Bx-PC3
Sorafenib	47.2 (43.7–51.0) -	76.1 (72.2–80.2) -	31.0 (28.4–33.7) -
+ β-Caryophyllene	16.1 (14.6–17.7) *** 2.9	43.9 (39.4–48.7) *** 1.7	5.8 (4.7–7.2) *** 5.3
+ β-Caryophyllene oxide	7.8 (6.8–8.9) *** 6.0	50.1 (43.7–56.8) ** 1.5	3.4 (2.8–4.2) *** 9.1
+ Verapamil	15.2 (11.8–19.3) *** 3.1	51.5 (46.1–57.3) ** 1.5	15.6 (13.9–17.6) ** 2.0
+ MK571	8.3 (6.1–11.2) *** 5.7	45.1 (37.5–53.8) ** 1.7	7.9 (6.5–9.5) *** 3.9

^a^ Reversal ratio (RR) represents the ratio between the IC_50_ values of sorafenib and its combination with β-caryophyllene, β-caryophyllene oxide, and the positive controls verapamil and MK571. ** *p* < 0.01 and *** *p* < 0.001 significantly lower than sorafenib IC_50_ value within the same cell line (Student’s *t*-test).

## Data Availability

Not applicable.

## Supplementary Materials

The following are available online at https://www.mdpi.com/article/10.3390/pharmaceutics14061264/s1, Figure S1: original image of western blotting analysis regarding STAT3 phosphorylation at tyrosine 705 residue (membrane 1), Figure S2: original image of western blotting analysis regarding total STAT3 (membrane 1), Figure S3: original image of western blotting analysis regarding STAT3 phosphorylation at tyrosine 705 residue (membrane 2), Figure S4: original image of western blotting analysis regarding total STAT3 (membrane 2).
