# Supplementary material for: Sorafenib Chemosensitization by Caryophyllane Sesquiterpenes in Liver, Biliary, and Pancreatic Cancer Cells: The Role of STAT3/ABC Transporter Axis
Source: Pharmaceutics. 2022 Jun 14;14(6):1264. doi: 10.3390/pharmaceutics14061264 (PMC9231089; doi:10.3390/pharmaceutics14061264)
Supplement: Supplementary file 1 [file pharmaceutics-14-01264-s001.zip › pharmaceutics-1730044-supplementary.pdf]

pharmaceutics

MDPI

Article

Sorafenib Chemosensitization by Caryophyllane Sesquiterpenes in Liver, Biliary, and Pancreatic Cancer Cells: The Role of STAT3/ABC Transporter Axis

Silvia Di Giacomo [1,*], Marco Gullì [1], Roberta Facchinetti [1], Marco Minacori [2], Romina Mancinelli [3], Ester Percaccio [1], Caterina Scuderi [1], Margherita Eufemi [2] and Antonella Di Sotto [1,*]

[1] Department of Physiology and Pharmacology "V. Erspamer", Sapienza University of Rome, P.le Aldo Moro 5, 00185 Rome, Italy; marco.gulli@uniroma1.it (M.G.); roberta.facchinetti@uniroma1.it (R.F.); ester.percaccio@uniroma1.it (E.P.); caterina.scuderi@uniroma1.it (C.S.)

[2] Department of Biochemical Science "A. Rossi Fanelli", Sapienza University of Rome, P.le Aldo Moro 5, 00185 Rome, Italy; marco.minacori@uniroma1.it (M.M.); margherita.eufemi@uniroma1.it (M.E.)

[3] Department of Anatomical, Histological, Forensic and Orthopedic Sciences, Sapienza University of Rome, P.le Aldo Moro 5, 00185 Rome, Italy; romina.mancinelli@uniroma1.it

* Correspondence: silvia.digiacomo@uniroma1.it (S.D.G.); antonella.disotto@uniroma1.it (A.D.S.)

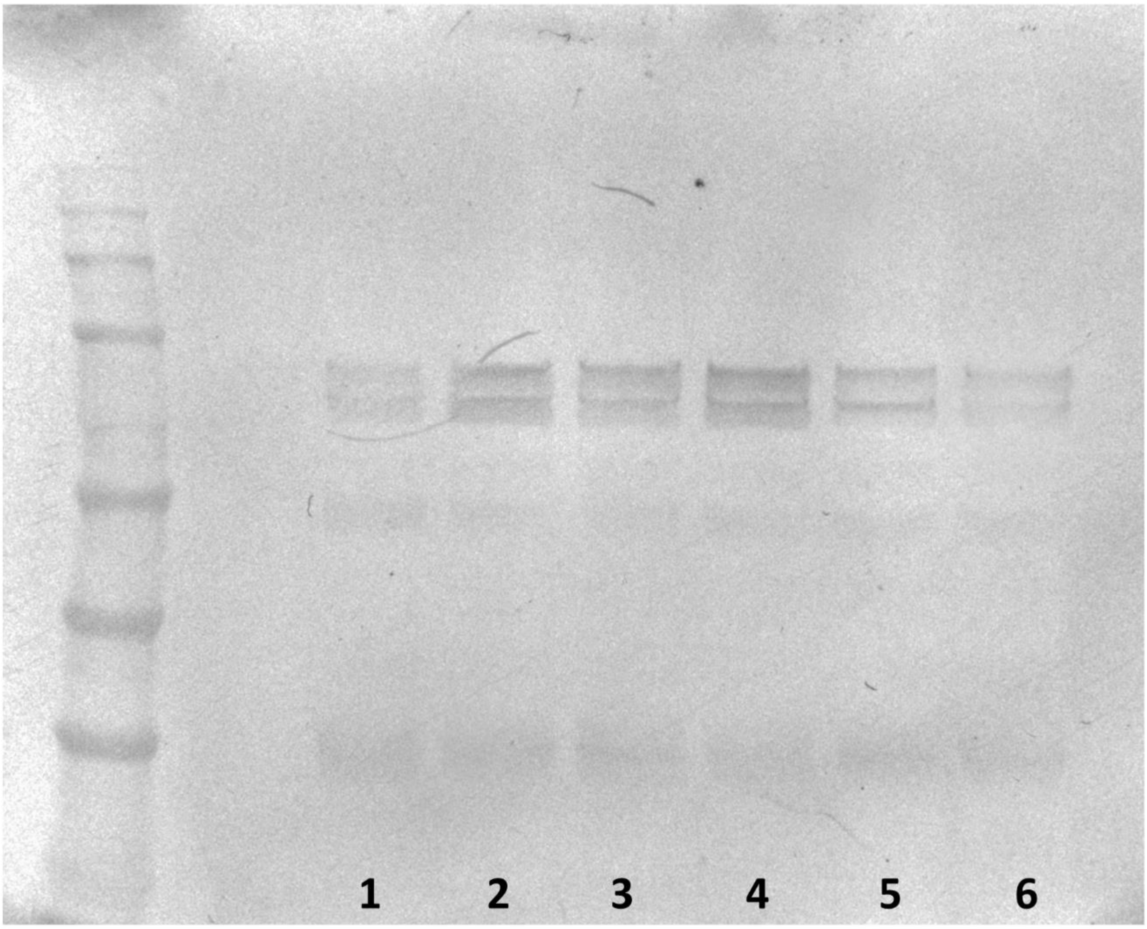

Figure S1. Original image of western blotting analysis regarding STAT3 phosphorylation at tyrosine 705 residue (membrane 1). 1) Control; 2) β-caryophyllene 10 μg/mL; 3) β-caryophyllene oxide 10 μg/mL; 4) sorafenib 10 μg/mL; 5) β-caryophyllene 10 μg/mL + sorafenib 10 μg/mL; 6) β-caryophyllene oxide 10 μg/mL + sorafenib 10 μg/mL.

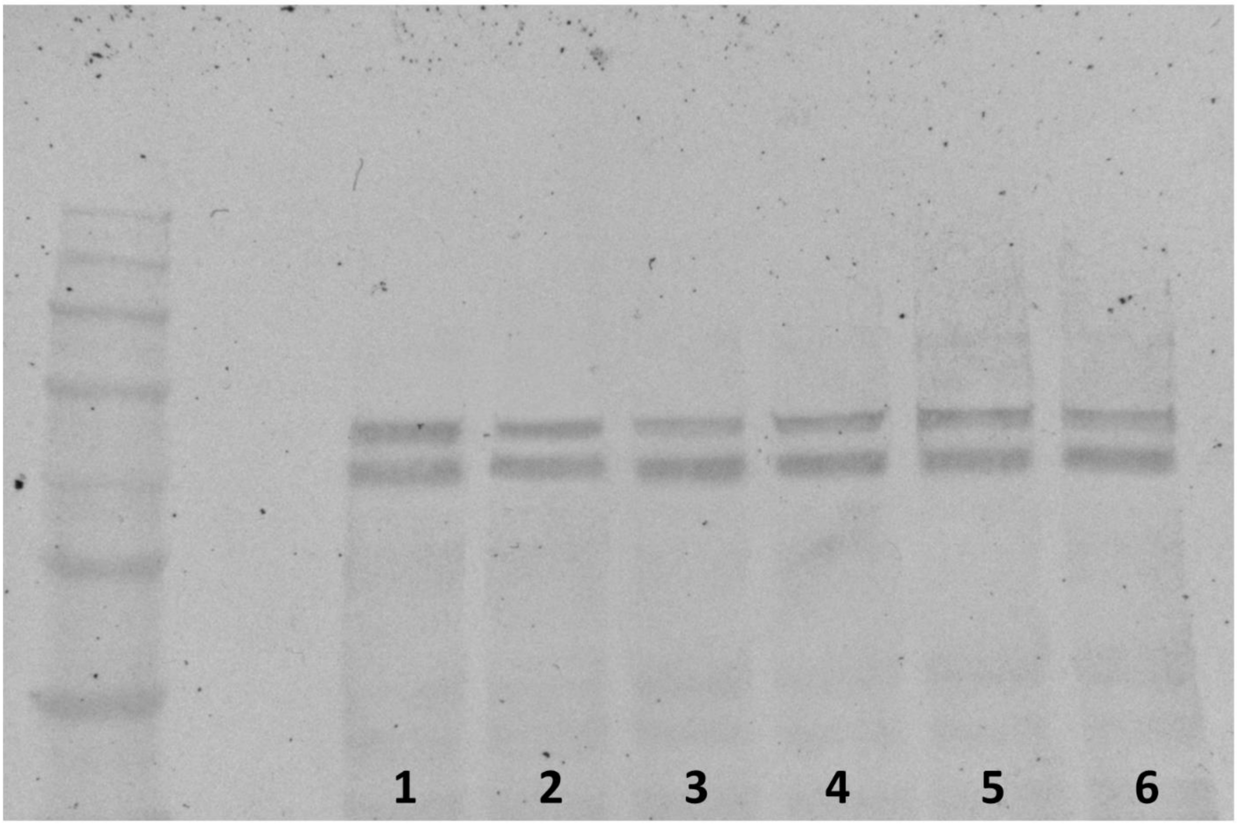

Figure S2. Original image of western blotting analysis regarding total STAT3 (membrane 1). 1) Control; 2) β-caryophyllene 10 μg/mL; 3) β-caryophyllene oxide 10 μg/mL; 4) sorafenib 10 μg/mL; 5) β-caryophyllene 10 μg/mL + sorafenib 10 μg/mL; 6) β-caryophyllene oxide 10 μg/mL + sorafenib 10 μg/mL.

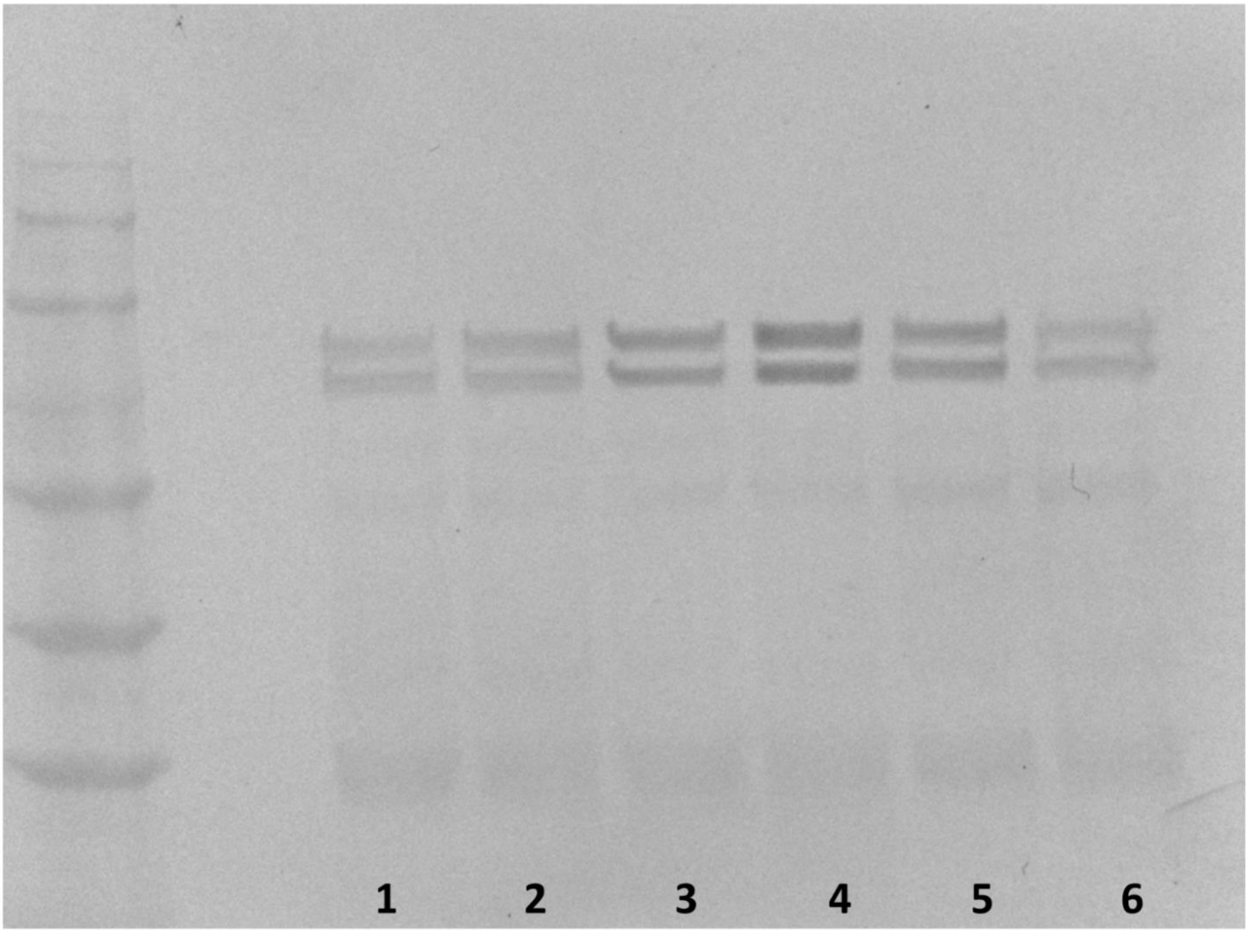

Figure S3. Original image of western blotting analysis regarding STAT3 phosphorylation at tyrosine 705 residue (membrane 2). 1) Control; 2) Verapamil 1 µg/mL; 3) MK571 1 µg/mL; 4) Sorafenib 10 µg/mL; 5) Verapamil 1 µg/mL + sorafenib 10 µg/mL; 6) MK571 1 µg/mL + sorafenib 10 µg/mL.

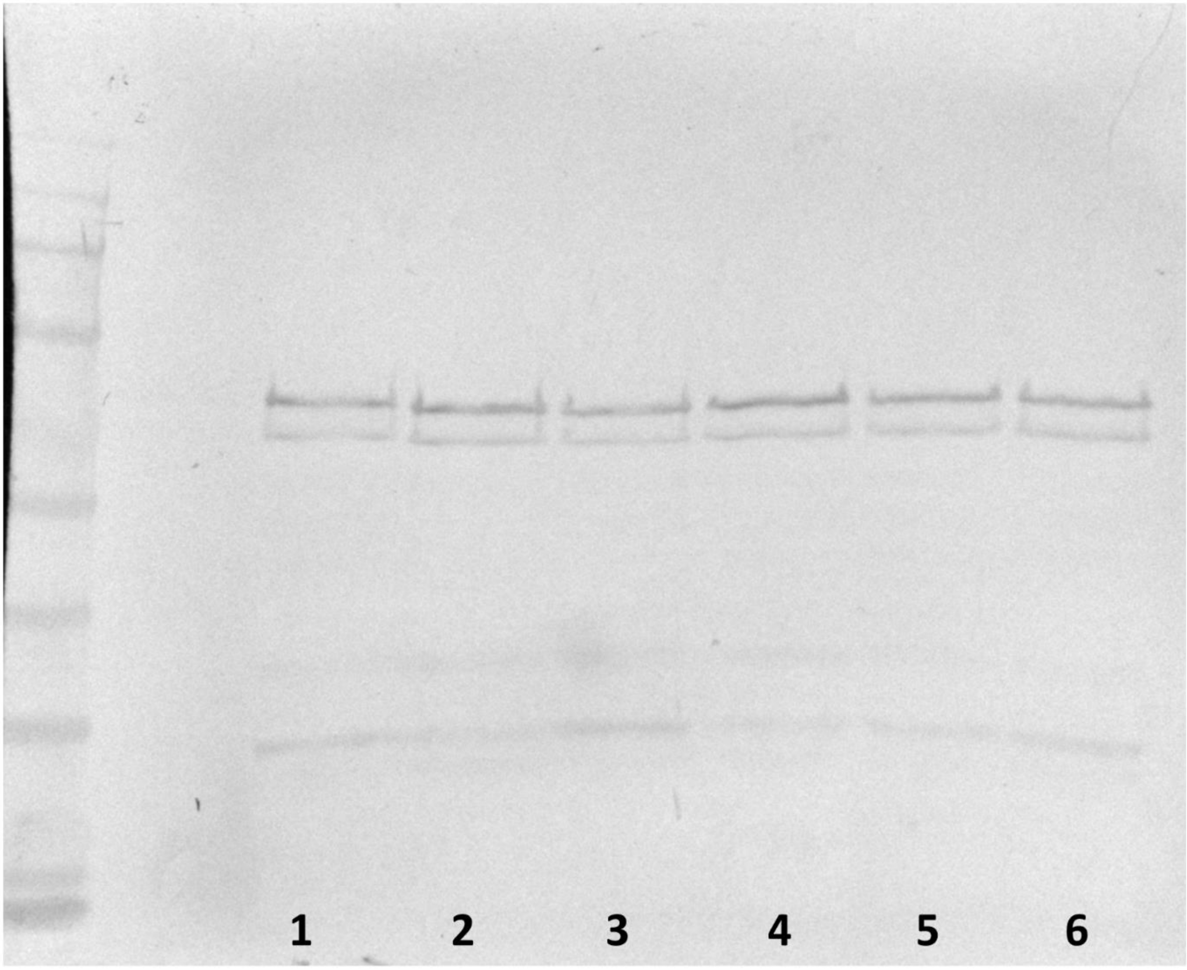

Figure S4. Original image of western blotting analysis regarding total STAT3 (membrane 2). 1) Control; 2) Verapamil 1 µg/mL; 3) MK571 1 µg/mL; 4) Sorafenib 10 µg/mL; 5) Verapamil 1 µg/mL + sorafenib 10 µg/mL; 6) MK571 1 µg/mL + sorafenib 10 µg/mL.
